# Bridging the gap between the economic evaluation literature and daily practice in occupational health: a qualitative study among decision-makers in the healthcare sector

**DOI:** 10.1186/1748-5908-8-57

**Published:** 2013-06-03

**Authors:** Johanna M van Dongen, Emile Tompa, Laurie Clune, Anna Sarnocinska-Hart, Paulien M Bongers, Maurits W van Tulder, Allard J van der Beek, Marieke F van Wier

**Affiliations:** 1 Body@Work, Research Center for Physical Activity, Work and Health, TNO-VU University Medical Center, van der Boechorststraat 7, 1081 BT Amsterdam, the Netherlands; 2 Department of Health Sciences and the EMGO + Institute for Health and Care Research, Faculty of Earth and Life Sciences, VU University Amsterdam, De Boelelaan 1085, 1081 HV Amsterdam, the Netherlands; 3 Department of Public and Occupational Health and the EMGO + Institute for Health and Care Research, VU University Medical Center, van der Boechorststraat 7, 1081 BT Amsterdam, the Netherlands; 4 Institute for Work & Health, 481 University Avenue, Suite 800, Toronto, Ontario, M5G 2E9, Canada; 5 Department of Economics, McMaster University, Kenneth Taylor Hall, Room 426, 1280 Main Street West, Hamilton, Ontario, L8S 4M4, Canada; 6 Dalla Lana School of Public Health, University of Toronto, 155 College Street Health Science Building, 6th floor, Toronto, Ontario M5T 3M7, Canada; 7 Faculty of Nursing, University of Regina, Education Building, Room 614, Regina, Saskatchewan, S4S 0A2, Canada; 8 TNO Healthy Living, Polarisavenue 151, 2132 JJ, Hoofddorp, the Netherlands; 9 Department of Epidemiology and Biostatistics and the EMGO + Institute for Health and Care Research, VU University Medical Center, van der Boechorststraat 7, 1081 BT Amsterdam, the Netherlands

**Keywords:** Occupational health and safety, Interventions, Decision-making process, Information needs, Evidence-based practice

## Abstract

**Background:**

Continued improvements in occupational health can only be ensured if decisions regarding the implementation and continuation of occupational health and safety interventions (OHS interventions) are based on the best available evidence. To ensure that this is the case, scientific evidence should meet the needs of decision-makers. As a first step in bridging the gap between the economic evaluation literature and daily practice in occupational health, this study aimed to provide insight into the occupational health decision-making process and information needs of decision-makers.

**Methods:**

An exploratory qualitative study was conducted with a purposeful sample of occupational health decision-makers in the Ontario healthcare sector. Eighteen in-depth interviews were conducted to explore the process by which occupational health decisions are made and the importance given to the financial implications of OHS interventions. Twenty-five structured telephone interviews were conducted to explore the sources of information used during the decision-making process, and decision-makers’ knowledge on economic evaluation methods. In-depth interview data were analyzed according to the constant comparative method. For the structured telephone interviews, summary statistics were prepared.

**Results:**

The occupational health decision-making process generally consists of three stages: initiation stage, establishing the need for an intervention; pre-implementation stage, developing an intervention and its business case in order to receive senior management approval; and implementation and evaluation stage, implementing and evaluating an intervention. During this process, information on the financial implications of OHS interventions was found to be of great importance, especially the employer’s costs and benefits. However, scientific evidence was rarely consulted, sound ex-post program evaluations were hardly ever performed, and there seemed to be a need to advance the economic evaluation skill set of decision-makers.

**Conclusions:**

Financial information is particularly important at the front end of implementation decisions, and can be a key deciding factor of whether to go forward with a new OHS intervention. In addition, it appears that current practice in occupational health in the healthcare sector is not solidly grounded in evidence-based decision-making and strategies should be developed to improve this.

## Background

The extent to which organizations allocate their limited resources towards occupational health and safety interventions (OHS interventions), including both worksite health promotion and health and safety interventions, is driven by some combination of legal, financial, and moral factors [1,2]. Among others, information on the costs and consequences of these interventions is therefore likely to be a valuable input into the decision of whether or not to implement or continue them. This is of particular importance in the healthcare sector, where OHS interventions focused on workers may be seen as redirecting resources away from higher priority ones more focused on patient care [3]. Furthermore, rising healthcare expenditures, experienced by many developed countries, may pose another limitation to the resources available for OHS interventions in the healthcare sector [4,5].

To aid occupational health decision-makers, different types of economic evaluations are carried out*.* Cost-benefit analyses (CBAs), also known as return-on-investment analyses, are conducted to provide insight into the net financial benefit or financial return by comparing incremental costs to incremental financial benefits of alternatives [6-9]. Cost-effectiveness analyses (CEAs) are conducted to provide insight into the incremental costs of an intervention per additional unit of effect gained. In cost-utility analyses (CUAs), the incremental costs of an intervention are compared to its attributable health improvements measured in utilities (*e.g.*, ‘quality adjusted life years’) [6].

During the last two decades, a growing number of articles has been published about the financial implications of OHS interventions [10], but their use and impact on day-to-day decision-making has not been adequately explored. However, as research indicates that results of economic evaluations of healthcare interventions for patients are rarely used among medical decision-makers [11-14], the use of economic evaluations among occupational health decision-makers is likely to be limited as well. Within the framework of evidence-based decision-making, it is essential that lessons learned from research are applied in practice. That is, continued improvements in occupational health can only be established if (implementation) decisions are based on the best available evidence. To ensure that this is the case, scientific evidence should meet the information needs of decision-makers. Specifically, disparities should be minimized between the way in which evidence is developed and presented and the way in which it is understood and used in daily practice [15]. In addition, because a lack of expertise in health economics (specifically economic evaluation) was found to be an important barrier to the use of economic evaluations among medical decision-makers [11,13], it is of importance that occupational health decision-makers are equipped with an adequate skill set to interpret and use scientific evidence on the financial implications of OHS interventions.

Until now, studies have been undertaken to gain insight into evidence-based decision-making and possible ways to improve it among occupational health professionals (*e.g.*, physicians, nurses) [16-18] and individual workers [19], but not among occupational health decision-makers. Therefore, as a first step in bridging the gap between the economic evaluation literature on OHS interventions and daily practice, the present study aimed to explore four issues: the process by which occupational health decisions are made; the importance given to the financial implications of OHS interventions; the sources of information used during the decision-making process; and occupational health decision-makers’ knowledge about different economic evaluation methods.

## Methods

In-depth interviews with occupational health decision-makers in the Ontario healthcare sector were conducted to explore the process by which occupational health decisions are made and the importance given to the financial implications of OHS interventions. Structured telephone interviews were conducted to explore the sources of information used during the decision-making process and occupational health decision-makers’ knowledge on economic evaluation designs. A qualitative approach was chosen, as little is currently known about these topics. Core categories of analytic foci have not yet been identified [20].

The present study was undertaken in collaboration with partners from the following organizations: the Public Services Health and Safety Association, the Ontario Nurses’ Association, and the Ontario Hospital Association. At three meetings held over the course of the study, partner representatives provided input and feedback on data collection activities.

### Ontario’s occupational health and safety and healthcare system

Canada is a federation of ten provinces and three territories. As such, labour legislation and healthcare are provincial and territory level jurisdictions. Therefore, the OHS system (including regulation and insurance) and the healthcare system vary somewhat between provinces/territories, though there are many common features [21]. In Ontario, regulatory responsibilities for the inspection and enforcement aspects of OHS lie with the ‘Ministry of Labour’ (MOL). Workers’ compensation is administered by the ‘Workplace Safety and Insurance Board’ (WSIB), a monopoly, not-for-profit insurance provider that covers approximately 70% of Ontario’s workforce. The WSIB is financed by payroll taxes levied on employers, with some variation among industries reflecting their different risk levels and accident experiences (*i.e.*, industry specific rate groups). Within these rate groups, financial incentives are administered for organizations through experience ratings. Organizations with better-than-average safety records receive a rebate, whereas those with a worse safety records are levied a surcharge [22]. The WSIB operates on a ‘no fault’ principle (*i.e.*, compensation is paid no matter who is at fault) and generally covers healthcare costs and lost earnings associated with occupational injury and disease [21,22]. Sickness absences that are not attributable to exposures at work are not compensable through workers’ compensation, though the universal, publicly-funded healthcare system provides medical services to all Ontario residents for needed care. Employers may provide wage-replacement benefits for these types of sickness absences. However, because these programs are not obligatory, only some employers offer them [21]. In the light of this study, it is also important to mention that workplaces with 20 or more employees are required by law to have a ‘Joint Health and Safety Committee’ (JHSC). A JHSC is made up of worker and employer representatives that work together to identify and resolve health and safety problems in their workplace [21].

Ontario’s universal, publicly-funded healthcare system is funded through transfer payments from the federal government and general taxes at the provincial level. Most hospitals are not-for-profit organizations that bill the ‘Ministry of Health and Long Term Care’ (MOHLTC) for a wide range of medically necessary services [21,23]. Long-term care (LTC), on the other hand, is provided by not-for-profit as well as for-profit facilities.

### Recruitment and sampling

In order to focus our sampling efforts and to keep the scope of the study manageable, a subset of organizations from the Ontario healthcare sector was selected, namely hospitals and LTC facilities. Participants for the in-depth interviews and structured telephone interviews were selected by means of purposeful sampling. This sampling method enables researchers to use their own judgement in order to select individuals who could provide in-depth information relevant to the research questions. Project partners assisted in identifying such individuals. Additionally, participants were selected by means of snowballing: *i.e.*, participants were asked whether they knew other people who they thought could provide relevant information about the occupational health decision-making process [20]. Participants had to be employees of an Ontario-based hospital or LTC facility that were either responsible for the daily occupational health operations or senior staff members. To reduce the risk of biased responses, decision-makers who participated in the in-depth interviews were excluded from participation in the structured telephone interviews. All participants were informed about the study purpose, were reassured of confidentiality, and provided written informed consent. Study details were approved by the University of Toronto’s Office of Research Ethics.

### In-depth interviews

In-depth interviews took place from June 2011 to August 2011 during an in-person or telephone meeting arranged at a time and location convenient to the participants. Interviews lasted on average 47 minutes (range: 12 to 116 minutes) and were conducted by two or three researchers (ET, AS-H, LC). One researcher was responsible for asking questions, whereas the other(s) took field notes and probed areas requiring more explanation. An interview protocol was used including questions and prompts. First, short questions were asked regarding the employment and workplace characteristics of the interviewee (*e.g.*, job description, years of relevant work experience, facility size). Subsequently, open-ended questions were asked to explore the decision-making process and the importance given to the financial implications of OHS interventions. The first open-ended question was ‘How does your organization go about starting and implementing an OHS intervention?’ Possible follow-up questions or prompts were ‘Can you describe how you evaluate OHS interventions?’ ‘What type of information helps move a plan forward?’ ‘How do you prioritize between alternatives?*’* ‘How does cost-benefit/cost-effectiveness fit into your decision-making process?’ Throughout the interview, participants were asked to illustrate their answers by giving examples of recent program implementation and/or continuation decisions, including those concerning both small versus large and mandated versus non-mandated OHS interventions. Among others, the participants’ examples concerned workplace violence, return to work, participatory ergonomics, and health education programs. Question prompts were slightly revised throughout the data collection process based on the researchers’ sense of what additional information would be useful and the participant’s position within the organization. The final topic list is provided in Additional file 1. Analytic field notes were written after each interview by one researcher (LC), including thoughts about the dynamics of the encounter and issues that may be relevant at the analytical stage [20]. All interviews were recorded and transcribed verbatim. After 15 interviews, the analytic field notes indicated that no new findings emerged (*i.e.*, data saturation). To be sure that data saturation was indeed reached, three additional interviews were conducted. As no new findings emerged from these interviews as well, data collection was terminated after 18 interviews.

### Data analysis: in-depth interviews

Data were analyzed using the constant comparative method, in which each item is checked or compared with the rest of the data to inductively establish analytical categories [24,25]. First, analytic field notes and transcripts were read to get a general understanding of the concepts under study and to get some insight into the dynamics of the interviews. Using Nvivo version 10 (QSR international, Burlington, USA), transcripts were subsequently open-coded by one researcher (JvD). That is, transcripts were read line by line and relevant passages were selected and coded, often by using the participants’ own words. Interview codes included both ‘descriptive’ (*i.e.*, within the immediate domain of the interview questions) and ‘analytic’ (*i.e.*, emerging and overarching) themes [20]. Throughout the coding process, conscious efforts were made to detect further examples of previously identified themes and, if applicable, to identify new ones [24-27]. Subsequently, similar codes were grouped into so-called analytical categories, and the analytical categories’ properties were explored as well as the relationships between those categories [25]. At various meetings held over the course of the data analysis process, identified codes, identified analytical categories, and interpretations of the data were checked and discussed with the interviewers (AS-H, ET, LC) to enhance the robustness of the findings. In all cases, consensus was reached through discussion.

### Structured telephone interviews

Structured telephone interviews were conducted by one researcher (AS-H) from November 2011 to February 2012 and lasted on average 27 minutes (range: 15 to 60 minutes). First, short questions were asked regarding the employment and workplace characteristics of the interviewee (*e.g.*, job description, facility size). Subsequently, participants were asked to what extent external sources of information were consulted when exploring whether a future intervention was worthwhile (*i.e.*, always, sometimes, never), and if so, what types of sources. Also, a list of inputs/costs and outcomes/consequences in economic evaluations of OHS interventions was provided to the participants, and they were asked to what extent these inputs/costs and outcomes/consequences were considered during the decision-making process (*i.e.*, always, sometimes, never). The list of inputs/costs and outcomes/consequences was derived from a previous study of one of the authors (unpublished data). Subsequently, participants were asked whether they were familiar with CBA, CEA, and CUA, and, if so, whether they could define these economic evaluation designs, whether they previously received training in economic evaluation-related topics, and whether they wanted to acquire more knowledge in this field. An overview of the structured interview items pertaining to research questions is provided in Additional file 2. All telephone interviews were recorded.

### Data analysis: structured telephone interviews

By listening to the audiotapes, descriptive statistics were prepared by two researchers (AS-H, JvD). Inputs/costs and outcomes/consequences of economic evaluations were regarded as ‘commonly considered’ if they were ‘always’ considered during the decision-making process by more than 50% of the participating healthcare facilities. Definitions of the various economic evaluation designs were scored as ‘correct’ if they included some variation of the following information: CBA, a comparison of costs and benefits, in which both are expressed in monetary terms; CEA, a comparison of costs and outcomes, in which costs are expressed in monetary terms and outcomes in natural units; and CUA, a comparison of costs and utilities, in which costs are expressed in monetary terms and utilities (*e.g.*, health improvements) in terms of ‘quality adjusted life years,’ or possibly some variant, such as ‘disability adjusted life years’ [6]. In all other cases, they were scored as ‘incorrect.’

## Results

### In-depth interviews

#### Participants

Eighteen in-depth interviews were conducted with a total of 19 participants (*i.e.*, one interview was conducted with two participants). Of them, 11 worked at a hospital and eight at a LTC facility. Twelve were female and seven male. Fifteen were responsible for the daily occupational health operations and four were senior staff members (Table 1).

**Table 1:** Characteristics of the study population

	**In-depth interviews**	**Structured telephone interviews**
**Participants [n.]**	**19**	**28**
**LTC [n. (%)]**	**8 (42)**	**1 (4)**
Female [n. (%)]	7 (88)	1(100)
Job description [n. (%)]		
OHS operations	6 (75)	1 (100)
Senior staff members	2 (25)	0 (0)
Years of relevant work experience [mean (SD)]	16.6 (7.8)	N.A.
**Hospital [n. (%)]**	**11 (58)**	**27 (96)**
Female [n. (%)]	5 (46)	21 (78)
Job description [n. (%)]		
OHS operations	9 (81)	26 (96)
Senior staff members	2 (19)	1 (4)
Years of relevant work experience [mean (SD)]	7.6 (2.8)	N.A.
**Interviews [n.]**	**18**	**25**
**LTC [n. (%)]**	**7 (39)**	**1 (4)**
Size [n. (%)]		
<250 employees	3 (43)	0 (0)
250-999 employees	4 (57)	1 (100)
Type [n. (%)]		
Public (not for profit)	4 (57)	1 (100)
Private (for profit)	3 (43)	0 (0)
**Hospital [n. (%)]**	**11 (61)**	**24 (96)**
Size [n. (%)]		
<250 employees	0 (0)	3 (13)
250-999 employees	3 (27)	6 (25)
1000-1999 employees	1 (9)	5 (21)
2000-9999 employees	5 (46)	7 (29)
>10000 employees	2 (18)	3 (13)
Type [n. (%)]		
Public (not-for-profit)	11 (100)	24 (100)
Private (for-profit)	0 (0)	0 (0)

Abbreviations: *n* number, *OHS* Occupational Health and Safety, *LTC* Long-Term Care facility, *N.A.* Not Available.

#### The process by which occupational health decisions are made

**Figure 1:**
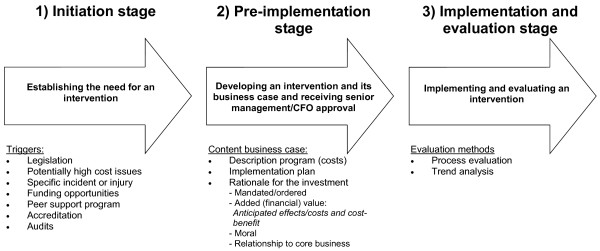
**Stages of the occupational health decision-making process.** Abbreviations: CFO Chief Financial Officer.

In general, the process by which occupational health decisions are made can be subdivided into three stages: initiation stage, pre-implementation stage, and implementation and evaluation stage (Figure 1).

#### Initiation stage

During the first stage of the decision-making process, the need for an intervention is mostly established by employees responsible for the daily occupational health operations and is generally triggered by one or more of the following factors:

##### Legislation

Legislation is given top priority and the implementation of many interventions is driven by regulatory requirements. As one participant noted, ‘First and foremost, obviously there’s the result of legislation … that we have to act upon.’ Legislation, however, only relates to health and safety interventions and not to worksite health promotion programs.

##### Potentially high cost issues

The need for an intervention may also be triggered by potentially high cost issues within the healthcare facility or sector. Internal statistics, such as incident report trends and sick leave data, are collected in all facilities through a variety of methods such as note taking and various software applications. In reviewing the acquired data, priority is given to finding ways to reduce costs through identifying high risk injury types or high exposure settings. As one participant noted, ‘We do collect incident injury data, employee injury data monthly.… We then put it into a quarterly graph and look at possible trends … a lot of initiatives are based on the incident trends.’

Some facilities benchmark these statistics against those of similar facilities to help put them into perspective. High cost issues within the facility are also identified by conducting on-site risk assessments and needs assessments among employees. External reports and scientific evidence are consulted by some facilities to identify high cost issues within the healthcare sector.

##### Specific incident or injury

After a specific incident or injury, interventions might be requested by the JHSC and/or senior management or ordered by the MOL. MOL orders are the result of so-called ‘significant incidents’ (*i.e.*, an employee is critically injured or killed at the workplace) that organizations have to respond to after MOL inspection. As one participant noted, ‘…that [intervention] came about because of an order from the Ministry of Labour. We had a worker that … fractured her arm. It was a critical injury, so we got the order.’

##### Funding opportunities

Occasionally, the federal or provincial government provides funding opportunities for OHS interventions. Many facilities apply for such grants, as is indicated by the following quote: ‘The government provided funding and, of course, we jumped on it like everybody else.’ Facilities have to spend these grants on a specific type of intervention or the reduction of a specific adverse health or safety outcome (*e.g.*, workplace violence). Another way in which facilities make use of funding opportunities is by participating in external research projects.

##### Peer support program

In Ontario, healthcare facilities, as well as other types of organizations, may participate in peer-support programs called Safety Group Programs. This is a performance-based rebate program developed by the WSIB. Organizations can join a safety group consisting of their peers to learn more from each other’s occupational health experiences. In the program, they are obliged to identify and implement five selected OHS interventions each year. A discount on insurance premiums is given for participating in these groups. Additionally, as one might expect, the successful implementation of OHS interventions may have positive implications for their insurance premiums, given that premiums are experience rated [28].

##### Accreditation

The need for an OHS intervention is sometimes identified during the hospital accreditation process. As one participant noted, ‘Initiatives come through quality improvement that we deal with through our annual accreditation processes.’ While not mandatory, almost all of Ontario’s hospitals and LTC facilities opt to go through regular accreditation reviews. The accreditation process is intended to ensure that healthcare facilities are meeting a common set of standards. Accreditation occurs on a three-year cycle and includes the measurement of various performance indicators (*e.g.*, patient safety and quality of care, infection prevention and control, medication management, organizational culture) [29].

##### Audits

OHS interventions are sometimes triggered by (upcoming) internal (*e.g.*, by the JHSC) or external audits (*e.g.*, by the MOL inspectorate). External audits may result in orders, which oblige a facility to address particular health hazards within a particular time period. With extreme health hazards, and repeated violations, a financial penalty may be imposed.

#### Pre-implementation stage

The second stage of the decision-making process, is generally characterized by the development of the intervention as well as its business case in order to receive approval for its implementation from senior management.

Based on the previously identified need(s), interventions are developed by employees responsible for the daily occupational health operations in consultation with various external (*e.g.*, similar facilities, safety group, consultants) and internal (*e.g.*, JHSC) sources of information. Sometimes, a small on-site pilot study is conducted to compare various program options, especially in the case of equipment purchasing decisions. Depending on its size, interventions are either developed by one person or a working group.

In most cases, senior management approval is needed before an intervention may be implemented. To convince them of the importance of a specific intervention, a so-called ‘business case’ is developed. These business cases generally include one or more of the following items: a description of the program and its costs, and sometimes that of alternatives, a program implementation plan, and a rationale for the investment. Various types of rationales emerged from the data (note that these rationales are linked to the triggers of OHS interventions, except for the moral rationale):

##### Mandated/ordered

The facility has to implement a certain intervention to comply with legislation, to deal with a specific incident (*e.g.*, after a MOL order), or to meet accreditation standards. As one participant noted, ‘They [senior management] always want to know, well, do we have to do it?’.

##### Added (financial) value

Implementation of the intervention may produce added value to the facility. In some cases this value is financial. For example, implementation may reduce the incidence of high cost issues (*e.g.*, high cost accidents, sick leave), leading to a worker’s compensation insurance rebate, or reduction in replacement staff costs. Implementation may also improve a healthcare facility’s reputation which, in turn, may affect its staff recruitment and retention abilities as well as its ability to raise charitable funds.

##### Moral

The intervention may be implemented for moral reasons. As one participant noted, ‘We actually go through the moral imperative about why it is not appropriate to injure people.’

##### Relationship to core business

Implementation of the intervention may improve the core business of the healthcare facility, namely patient care. Participants specifically indicated that they made the connection between OHS interventions and patient care, because their facility receives funds for the provision of patient care activities and not directly for occupational health.

As part of the ‘added (financial) value’ rationale, an overview of the anticipated effects, benefits, and/or cost-benefit are often presented. Most participants indicated that ex-ante CBAs formed the basis of a business case, but these cases are very high level and stylized in nature. That is, they are not supported by rigorous internal statistics and/or scientific evidence. To illustrate, one participant described the content of a CBA as follows:

‘Maybe the costs with the WSIB, the modified work etcetera … may have been $60,000 for the year versus the cost of equipping the unit, which would have been maybe $10,000 or $12,000. And so, obviously, we wanna do something like that.’

This finding is also supported by the following comment of a participant with work experience in both the private and public sector:

‘I know in the private sector when we were doing a cost-benefit analysis on the purchase of a piece of equipment, it was much more quantitative …. here, it seems to be a little more subjective and I don't really understand why that is.’

Cost-comparison analyses of various program options are also performed to identify the least costly alternative, but these analyses are mainly conducted for mandated interventions.

After completion, business cases are taken forward to senior management for approval. In most cases, a final decision is made in consultation with the chief financial officer, especially in the case of expensive interventions. The specific strategies used by the senior management to make and prioritize occupational health decisions are not transparent. Most operational personnel were unclear about the process, while others described it as subjective. However, the approval process for mandated/ordered interventions and those that require minimal financial investments is less demanding (in terms of information and time required to make the business case) than that of non-mandated and more costly ventures and they are therefore more quickly approved. In LTC facilities, the approval process is not always as complex as described above. For example, when a need for an intervention is established, operational personnel may speak directly to the chief executive officer or director who has the ultimate responsibility for the organization. This is because LTC facilities are generally smaller than hospitals and have a flatter hierarchy.

#### Implementation and evaluation stage

During the third stage of the decision-making process, an OHS intervention is implemented and evaluated by performing a process evaluation and/or trend analysis. Process evaluations are generally aimed at exploring program execution, and employee satisfaction, compliance, attendance, and/or awareness. Process evaluation data is gathered through surveys, observations, and/or verbal feedback. Trend analyses are conducted to get an indication of the intervention’s effectiveness. Therefore, various intervention-related measures, such as accident frequency or sickness absence rates, are collected from company records. Analyses explore whether their frequency decreased after implementation. Some participants, however, doubted the validity of results; either the integrity of their data, or concerns that observed trends were caused by factors other than the intervention. The latter is evident from the following comment:

‘So overall, we did see a reduction, but it's hard to say whether or not that reduction was because the weather had gotten warmer or because it was just a coincidence. We're not too sure yet.’

Most participants indicated that far from all interventions are subjected to such evaluations and that ex-post CBAs are generally not performed. Their most important explanation for this was that they lacked the resources (time, money, and ability) to do so. As one participant noted, ‘The reason why we don’t do those evaluations on an ongoing basis is because it would cost money to do so.’ Other explanations were: lack of good data, and lack of economic evaluation skills.

#### The importance given to the financial implications of OHS interventions

Almost all participants indicated that information on the financial implications of OHS interventions is of great importance during the decision-making process, especially their cost-benefit. This is due to the fact that investing in those kinds of interventions literally affects a healthcare facility’s ability to provide patient care, as they have a tight budget (even the for-profit LTC facilities) and all occupational health expenses appear to take away from the patient care budget. Another reason for its importance is that healthcare facilities are mostly publicly funded. As one participant noted, ‘What makes this industry very different is the object. The politics, the perception that, because this is publicly funded…., the need not to waste is greater than on the other side.’ Information on the financial implications of mandated/ordered interventions seems less important. As one participant noted, ‘For our other health and safety programs, really, I would say that the only cost-benefit is that we don't get fined.’

### Structured telephone interviews

#### Participants

Twenty-five structured telephone interviews were conducted with a total of 28 participants. Of them, 27 worked at a hospital and one at a LTC facility. Twenty-two interviews were conducted with one participant and three with two participants. Twenty-two were female and six male. Twenty-seven were responsible for the daily occupational health operations and one was a senior staff member (Table 1).

#### The (sources of) information used during the occupation health decision-making process

##### Sources of information

To explore whether a future intervention is worthwhile, external sources of information were ‘always’ consulted during the decision-making process at 10 facilities (40%), ‘sometimes’ at 13 (52%), and ‘never’ at two (8%). Peer healthcare facilities were the principal external source of information (n = 23; 92%) and were either contacted directly or via a Safety Group Program. At five facilities (20%), participants indicated that they searched for scientific evidence on programs similar to those under consideration for implementation. Other external sources of information were: employers’ associations (28%), the government (MOL/MOHLTC)(20%), the WSIB (20%), vendors (8%), law firms (4%), safety specialists (4%), and unions (4%).

##### Inputs/costs and outcomes/consequences considered during the decision-making process

A broad range of inputs/costs was considered during the decision-making process, though hard cost items (*e.g.*, cost of equipment purchases, equipment installation, employee training) were more commonly considered than softer cost items (*e.g.*, cost of administration, planning, promotion, and evaluation). This was mainly due to the fact that the latter were often considered as part of the regular day-to-day responsibilities of the affected departments (Table 2). A broad range of outcomes/consequences was considered as well. The number of injuries, illnesses, and sickness absences were considered at all facilities. Other commonly considered outcomes/consequences were days lost due to injuries or illnesses, accommodating injured or ill workers, quality of care and patient safety, employer workers’ compensation insurance premiums, and meaningful return to work. In contrast, items such as impact on productivity (*i.e.*, presenteeism), attraction and retention (*i.e.*, turnover), worker replacement expenses, and labour relations climate were less commonly considered (Table 2).

**Table 2:** Inputs/costs and outcomes/consequences considered during the decision-making process

**Items**	**How often are these items considered during the decision-making process*?***		
**Inputs (Costs)**	**Always**	**Sometimes**	**Never**
	**[n. (%)]**	**[n. (%)]**	**[n. (%)]**
Health and safety staff time	11 (44)	10 (40)	4 (16)
Training the worker	15 (60)	10 (40)	0 (0)
Planning, promotion and evaluation	7 (28)	12 (48)	6 (24)
Equipment purchases	23 (92)	2 (8)	0 (0)
Administration	6 (24)	14 (60)	5(20)
Equipment installation	17 (68)	8 (32)	0 (0)
Ongoing equipment repair and maintenance	12 (48)	10 (40)	3 (12)
Professional / consultant fees	18 (72)	5 (20)	2 (8)
Ongoing supplies	14 (56)	10 (40)	1 (4)
**Outcomes (Consequences)**	**Always**	**Sometimes**	**Never**
	**[n. (%)]**	**[n. (%)]**	**[n. (%)]**
Number of injuries, illnesses, sickness absences	25 (100)	0 (0)	0 (0)
Days lost due to injuries, illnesses, and sickness absences	22 (88)	2 (8)	1 (4)
Quality of care and patient safety	16 (64)	7 (28)	2 (8)
Attraction and retention	7 (28)	16 (64)	2 (8)
Accommodating injured or ill workers^1^	14 (56)	10 (40)	1 (4)
Impact on productivity	12 (48)	12 (48)	1 (4)
Worker replacement expenses	10 (40)	11 (44)	4 (16)
Employer workers’ compensation insurance premiums	15 (60)	7 (28)	3 (12)
Employer claims management expenses	11 (44)	9 (36)	5 (20)
Overtime payment	8 (32)	12 (48)	5 (20)
Meaningful return to work^2^	14 (58)	8 (33)	2 (8)
Labour relations climate^2^	12 (50)	11 (46)	1 (4)

Abbreviations: *n.* number.

^1^Provision of accommodated work to injured workers to reduce the duration of work absence.

^2^One participant did not answer this question, as he/she was unsure.

#### Occupational health decision-makers’ knowledge of different economic evaluation designs

Most participants (93%) were familiar with the concept of CBA and many (72%) were able to give a correct definition. For them, it meant comparing the costs of implementing an intervention with the financial consequences it was expected to bring:

‘It’s where you factor in all the costs of the intervention … Direct costs associated with whatever it is that you are trying to purchase … On the benefit side you would still put it into dollars, but it would be attributing things like reduced sick time and reduced injury costs. So both sides of the equation and then you would come out with … a positive or negative return on your investment.’

CBAs were undertaken at most facilities (92%), and formed the basis of a business case. These analyses were generally performed from the employer’s perspective and not from the worker’s or societal perspective.

Most participants (71%) indicated that they were familiar with the concept of CEA, but few (11%) were able to give a correct definition. Most of them thought it to be synonymous with on-going monitoring and evaluation and not necessarily a comparison of costs with outcomes measured in natural units:

‘Cost-effectiveness is looking at how effective an initiative is in terms of … is the outcome what we anticipated it to be.’

‘Looking at the outcomes to determine whether what you anticipated to be the expected outcome, did you really reach those … But it’s a complete guess.’

Others thought it to be synonymous to CBA:

‘Is the investment of money and time worth the effort and that we will have a return on investment.’

Most participants were not familiar with the concept of CUA; only one indicated that he had heard of it, but was not able give a correct definition. Some participants tried to guess the definition, but most of them thought it to be an evaluation of the utilization (uptake) of an intervention:

‘Well utility is utilization, so I guess.... if we spent 25,000 Dollars..... We want to know whether they [the new equipment] are actually being used.’

Few participants (36%) received training in an area related to economic evaluations, such as a business proposal course, certified accountant training for financial planning, and business case sessions on program evaluations. When asked whether they were interested in receiving training, 79% (n = 22) expressed interest, 18% (n = 5) were not interested, and one (4%) was uncertain. Of those not interested, lack of interest was expressed because they already considered themselves familiar with economic evaluation methods, were already adequately skilled at making informed decisions, or they considered their facility too small for such training to be of added value. Participants who expressed interest in receiving training felt that it would provide them with the skills required to make more informed implementation decisions and to undertake better evaluations themselves.

When asked what topic they wanted to learn more about, most of the participants (77%) indicated that they wanted to acquire more knowledge on CBA and/or writing a business case. Some also indicated that they wanted to acquire more knowledge about CEA and CUA after these terms were briefly explained to them (CEA: 36%, CUA: 36%).

## Discussion

As a first step in bridging the gap between the economic evaluation literature and daily practice in health and safety, this study aimed to provide insight into the occupational health decision-making process and information needs of decision-makers in the Ontario healthcare sector. Results showed that this process can be subdivided into three stages: initiation stage, during which the need for an intervention is established; pre-implementation stage, during which an intervention and its business case are developed in order to receive senior management approval; and implementation and evaluation stage, during which an intervention is implemented and evaluated. In line with previous research [1,2], organizations were found to invest in OHS interventions for legal, financial, and moral reasons, and information on their financial implications was found to be of great importance to the decision-making process. Results also indicated that occupational health decisions are currently not being made in an evidence-based manner. That is, scientific evidence on the (financial) implications of OHS interventions was found to be rarely consulted and sound ex-post program evaluations were hardly ever performed [30-32]. Also, there seemed to be a need to advance the decision-makers’ economic evaluation skill set, as they were either not familiar with economic evaluation methods or had only a modest amount of training in this area. Therefore, strategies should be developed to overcome these issues.

### Strengths and limitations

Important strengths of the present study are its explorative and qualitative design. This enabled us to be one of the first to provide detailed insight into the extent to which occupational health decisions are made in an evidence-based manner, as well as to indentify the information needs of occupational health decision-makers. By simultaneously exploring both issues, we were able to provide some initial clues to occupational health researchers as to how they might better frame and disseminate their studies to ensure uptake in healthcare organizations as well as organizations in other sectors.

Several methodological limitations deserve attention as well. First, the present study was restricted to a single industry, in a single region of one country. This was done to keep the scope of the study manageable, but likely bears on the generalizability of its results. For example, one might expect that occupational health decisions are made differently in sectors where budgets for occupational health are less tight. Furthermore, occupational health decision-making processes likely vary between jurisdictions (*e.g.*, countries with different OHS and/or healthcare systems), in particular regarding the triggers of OHS interventions. Therefore, future studies should be conducted to explore the extent to which the present findings are generalizable beyond the healthcare sector and beyond Ontario, Canada. Second, due to the qualitative design of the present study, a limited number of interviews were conducted. However, as the healthcare facilities represented by the participants, in aggregate, employ a large number of Ontario healthcare workers, the extent to which this reduced the external validity of the present findings is probably small. Third, data were obtained through interviews, which may have caused ‘social desirability bias.’ For example, because participants were aware of the fact that they were interviewed by occupational health researchers, they may have overstated their use of scientific evidence as well as the quality of their decision-making process.

### Improving evidence-based practice in occupational health

Sackett *et al.* (2000) identified two separate stages for evidence-based practice. The first stage concerns the generation of scientific evidence and relies heavily on the academic body of a profession. The second stage concerns the use of scientific evidence into daily practice [33,34]. To improve the quality of the occupational health decision-making process, both stages should be addressed.

When generating scientific evidence, occupational health researchers should ensure that their products are in line with the information needs of occupational health decision-makers, as it is unrealistic to expect decision-making processes to be redesigned around research priorities [15,35]. The present study provided some initial clues as to what these information needs are. For example, process evaluation data and information on the interventions’ impact on corporate reputation and business results were found to be of interest to decision-makers. In addition, CBAs performed from the employer’s perspective formed the basis of business cases for occupational health. Within these analyses, hard cost items (*e.g.*, equipment costs, employee training costs) were of particular importance and benefits were commonly expressed in terms of reduced injury-, illness-, sickness absence-, and/or workers’ compensation-related costs. In line with previous research [2], data on staff retention and productivity were considered relevant but not commonly used. The latter could probably be explained by the fact that these types of benefits are generally viewed as harder to identify and hard to monetize. Researchers, especially those conducting clinical trials, should be encouraged to report on the employer’s cost-benefit of OHS interventions as well as their impact on corporate reputation and business results. This, however, does not negate the value of other types of economic evaluations. Various potential program benefits (*e.g.*, job satisfaction, corporate reputation) and health outcomes are hard to monetize and may therefore not be included in a CBA. A possible way to deal with these so-called ‘intangible benefits’ is to conduct a CEA to estimate the incremental costs per ‘intangible benefit’ gained [8]. In addition, the adoption of the societal perspective may provide insight into the distribution of costs and benefits between various stakeholders and thereby allows for bargaining between them [6]. The latter is of particular importance in countries with universal healthcare coverage or dual-payer systems, because employers bear most of the costs of OHS interventions, while the government and/or healthcare system reaps a large part of its benefits (*i.e.*, reduced medical spending) [1].

In daily practice, decisions have to be made within a limited time frame and many decision-makers lack the skills to determine what evidence is most reliable, and what evidence should be considered, under which circumstances [36]. It is therefore advisable to provide busy decision-makers with critical summaries of published studies [37]. Within the occupational health research field, systematic reviews are increasingly being conducted to critically appraise and summarize the current evidence on the (financial) implications of various OHS interventions. These systematic reviews, however, do not seem to be used in daily practice. This is probably due to the fact that many decision-makers lack the time and skill set required to read and understand these systematic reviews as well. Additionally, most of these reviews are published in scientific journals not well known or inaccessible to occupational health decision-makers. Therefore, it is important to transmit systematic review results to decision-makers in easy-to-use formats [35]. This may be accomplished by publishing review fact-sheets in journals and newsletters more familiar to occupational health decision-makers and/or by distributing them through governmental institutes, employers’ associations, and workers’ compensation insurance boards. In addition, (more) best practice guidelines could be developed in which scientific evidence is summarized, and if unavailable, supplemented by expert opinions [36]. To improve evidence-based practice, it is also important to educate decision-makers about economic evaluation methods, as well as the need and importance of integrating scientific evidence into day-to-day occupational health decision-making processes. The former is of particular importance, as many decision-makers were not familiar with various economic evaluation designs, which may not only limit the use of such studies in daily practice, but may also lead to misinterpretations of their results. Occupational health decision-makers may be educated through a variety of formal and informal means, including the development of handbooks and workshops on economic evaluation methods and evidence-based practice, integrating these topics into management and/or occupational health training programs, and involving occupational health decision-makers in the process of commissioning studies [38,39]. Participation in scientific studies is namely closely linked with the uptake of their results [37] and may simultaneously lead to an enhanced economic evaluation skill set. Another option would be for researchers to develop hands on program evaluation software applications, so that decision-makers can conduct their own ex-ante or ex-post program evaluations in a relatively non-time consuming way. Additionally, more evidence is needed on the merits of evidence-based decision-making in occupational health, specifically, evidence that demonstrates that it improves organizations’ performance. More economic evaluations of OHS interventions are needed to build a solid evidence base in order to support evidence-based practices in occupational health [34].

### Implications for future research

Researchers, especially those conducting clinical trials, are recommended to report on the cost-benefit of OHS interventions from the employer’s perspective as well as other perspectives. The impact of OHS interventions on operational outcomes and corporate reputation are two important pieces of information of occupational health decision-making. In the healthcare field, patient outcomes are particularly important. In addition, future research should focus on the extent to which the present findings are generalizable to other jurisdictions and on the effectiveness of possible strategies to improve evidence-based decision-making in occupational health.

## Conclusion

This exploratory qualitative study on the occupational health decision-making process in healthcare suggests that the process generally consists of three stages; initiation stage: establishing the need for an intervention; pre-implementation stage: developing an intervention and its business case; and implementation and evaluation stage, implementing and evaluating an intervention. Organizations invest in occupational health for legal, financial, and/or moral reasons. Financial information is particularly important at the front end of implementation decisions, and can be a key deciding factor of whether to go forward with a new OHS intervention. In addition, it appears that current practice in occupational health in the healthcare sector is not solidly grounded in evidence-based decision-making and strategies should be developed to improve this.

## Abbreviations

CBA: Cost-benefit analysis; CEA: Cost-effectiveness analysis; CUA: Cost-utility analysis; JHSC: Joint health and safety committee; LTC: Long term care; MOHLTC: Ministry of health and long term care; MOL: Ministry of labour; OHS: Occupational health and safety; WSIB: Workplace Safety and Insurance Board.

## Competing interests

The authors declare that they have no competing interests.

## Authors’ contributions

ET and LC designed the study. ET, LC, and AS-H conducted the interviews. JvD, ET, LC, and AS-H participated in the data analysis process. JvD wrote the manuscript. All authors contributed to the data interpretation as well as the drafting of the manuscript. All authors read and approved the final manuscript.

## Supplementary Material

Additional file 1 Topic list of the in-dept interviews. Click here for file

Additional file 2 Topic list of the structured telephone interviews. Click here for file
