# Supplementary material for: Bridging the gap between the economic evaluation literature and daily practice in occupational health: a qualitative study among decision-makers in the healthcare sector
Source: Implement Sci. 2013 Jun 3;8:57. doi: 10.1186/1748-5908-8-57 (PMC3674944; doi:10.1186/1748-5908-8-57)
Supplement: Additional file 1 — Topic list of the in-dept interviews. [file 1748-5908-8-57-S1.doc]

**Additional file 1: Topic list of the in-dept interviews**

1. How does your organization go about starting and implementing an OHS intervention? You may think about something you recently did (small versus large and mandated versus non-mandated OHS interventions).

Prompts: What is the decision-making process? From where do the resources come?

2) Can you describe how you evaluate OHS interventions?

Prompts: How well resourced are you to evaluate such initiatives? Who is responsible? What type of information helps move a plan forward? What information do people draw upon for evaluation? What kind of data and information are available to you for evaluation? What other resources such as funds and occupational health staff are available to you? How do you prioritize between alternatives? How are OHS interventions approved? Who is responsible?

What kinds of things do you do after program implementation to monitor, evaluate, and assess whether you reached your targets?

3) How does cost-benefit/cost-effectiveness fit into your decision-making process?

Prompts: How does your workplace know if an OHS intervention is (financially) worthwhile? What kind of costing/evaluation does your workplace do beforehand? Do you do a business case/cost-benefit analysis? What kinds of outcomes are considered? Where do you get data for this?
