# Supplementary material for: Bridging the gap between the economic evaluation literature and daily practice in occupational health: a qualitative study among decision-makers in the healthcare sector
Source: Implement Sci. 2013 Jun 3;8:57. doi: 10.1186/1748-5908-8-57 (PMC3674944; doi:10.1186/1748-5908-8-57)
Supplement: Additional file 2 — Topic list of the structured telephone interviews. [file 1748-5908-8-57-S2.doc]

**Additional file 2: Topic list of the structured telephone interviews**

**Sources of information used during the occupational health decision-making process**

Sources of information

1. Can you list the kinds of information your workplace gathers to know if a future OHS intervention will be worthwhile?
2. Do you get information on outcomes (results) for a future OHS intervention from external sources? (Always; Sometimes; Never)
   1. If so, what types of sources?

Inputs/costs and outcomes/consequences considered during the decision-making process

1. I’m going to list a few inputs (costs) of economic evaluations of OHS interventions. I’d like you to tell me if you use this information in an evaluation.

|  | **Inputs / Costs** | **Ranking total** |
| --- | --- | --- |
| 1 | Health and safety staff time | always, sometimes, never |
| 2 | Training the worker | always, sometimes, never |
| 3 | Planning, promotion and evaluation | always, sometimes, never |
| 4 | Equipment purchases | always, sometimes, never |
| 5 | Administration | always, sometimes, never |
| 6 | Equipment installation | always, sometimes, never |
| 7 | Ongoing equipment repair and maintenance | always, sometimes, never |
| 8 | Professional / consultant fees | always, sometimes, never |
| 9 | Ongoing supplies | always, sometimes, never |

1. I’m going to list a few outcomes (consequences) of economic evaluations of OHS interventions. I’d like you to tell me if your organization considers them in an evaluation.

|  | **Outcomes / Consequences** | **Ranking total** |
| --- | --- | --- |
| 1 | Number of injuries, illnesses, sickness absences | always, sometimes, never |
| 2 | Days lost due to injuries, illnesses and general sickness | always, sometimes, never |
| 3 | Quality of care and patient safety | always, sometimes, never |
| 4 | Attraction and retention | always, sometimes, never |
| 5 | Accommodating injured or ill workers | always, sometimes, never |
| 6 | Impact on productivity | always, sometimes, never |
| 7 | Worker replacement expenses | always, sometimes, never |
| 8 | Employer workers' compensation insurance premiums | always, sometimes, never |
| 9 | Employer claims management expenses | always, sometimes, never |
| 10 | Overtime payments | always, sometimes, never |
| 11 | Meaningful return to work | always, sometimes, never |
| 12 | Labour relations climate | always, sometimes, never |

**Occupational health decision makers’ knowledge of different economic evaluation designs**

I’m going to list a few economic evaluation terms you may or may not know. I just want you to tell me if you have heard of them and what the terms mean to you. Not everyone knows the meaning of the terms so just tell me what you think it is.

1. Cost-benefit analysis
   1. What does it mean to you?
   2. Does your workplace perform them? (Always; Sometimes; Never)
2. Cost-effectiveness analysis
   1. What does it mean to you?
   2. Does your workplace perform them? (Always; Sometimes; Never)
3. Cost-utility analysis
   1. What does it mean to you?
   2. Does your workplace perform them? (Always; Sometimes; Never)
4. Have you or anyone at your workplace had any training course, education, or guidance in economic evaluation for OHS interventions? (Yes/No)
   1. What type?
   2. Where did you get it?
   3. How long was it? (hours, days, weeks)
5. Is there anything in particular that you feel you want/need to learn more about to do evaluations of OHS interventions? (Yes/No)
